# Carbapenem Resistance in Acinetobacter nosocomialis and Acinetobacter junii Conferred by Acquisition of *bla*_OXA-24/40_ and Genetic Characterization of the Transmission Mechanism between Acinetobacter Genomic Species

**DOI:** 10.1128/spectrum.02734-21

**Published:** 2022-02-09

**Authors:** Cristina Lasarte-Monterrubio, Paula Guijarro-Sánchez, Alba Bellés, Juan Carlos Vázquez-Ucha, Jorge Arca-Suárez, Carlos Fernández-Lozano, German Bou, Alejandro Beceiro

**Affiliations:** a Microbiology Department of the University Hospital A Coruña (CHUAC), Institute of Biomedical Research of A Coruña (INIBIC), CIBER de Enfermedades Infecciosas, A Coruña, Spain; b Microbiology Department of the University Hospital Arnau de Vilanova, Lleida, Spain; c Department of Computer Science and Information Technologies, Faculty of Computer Science, Research Center of Information and Communication Technologies (CITIC), Universidade da Coruña, A Coruña, Spain; University of Manitoba

**Keywords:** carbapenemase OXA-24/40, *Acinetobacter junii*, *Acinetobacter nosocomialis*, *Acinetobacter* species, carbapenem resistance, horizontal transfer, XerC/XerD sequences

## Abstract

Carbapenem resistance is increasing among Gram-negative bacteria, including the genus Acinetobacter. This study aimed to characterize, for the first time, the development of carbapenem resistance in clinical isolates of Acinetobacter junii and Acinetobacter nosocomialis conferred by the acquisition of a plasmid-borne *bla*_OXA-24/40_ gene and also to characterize the dissemination of this gene between species of Acinetobacter. Carbapenem-resistant A. nosocomialis HUAV-AN66 and A. junii HUAV-AJ77 strains were isolated in the Arnau de Vilanova Hospital (Spain). The genomes were sequenced, and *in silico* analysis were performed to characterize the genetic environment and the OXA-24/40 transmission mechanism. Antibiotic MICs were determined, and horizontal transfer assays were conducted to evaluate interspecies transmission of OXA-24/40. Carbapenems MICs obtained were ≥64 mg/L for HUAV-AN66 and HUAV-AJ77. Genome analysis revealed the presence in both strains of a new plasmid, designated pHUAV/OXA-24/40, harboring the carbapenem-resistance gene *bla*_OXA-24/40_ and flanked by sequences XerC/XerD. pHUAV/OXA-24/40 was successfully transferred from A. nosocomialis and A. junii to a carbapenem-susceptible A. baumannii strain, thus conferring carbapenem resistance. A second plasmid (pHUAV/AMG-R) was identified in both clinical isolates for the successful horizontal transfer of pHUAV/OXA-24/40. *bla*_OXA-24/40-_carrying plasmids of the GR12 group and showing high identity with pHUAV/OXA-24/40 were identified in at least 8 Acinetobacter species. In conclusion the carbapenemase OXA-24/40 is described for the first time in A. nosocomialis and A. junii. In both isolates the *bla*_OXA-24/40_ gene was located in the GR12 pHUAV/OXA-24/40 plasmid. GR12 plasmids are implicated in the dissemination and spread of carbapenem resistance among Acinetobacter species.

**IMPORTANCE** Acinetobacter baumannii is one of the most relevant pathogens in terms of antibiotic resistance. The main resistance mechanisms are the carbapenem-hydrolyzing class D β-lactamases (CHDLs), especially OXA-23 and OXA-24/40. In addition to A. baumannii, there are other species within the genus Acinetobacter, which in general exhibit much lower resistance rates. In this work we characterize for the first time two clinical isolates of Acinetobacter nosocomialis and Acinetobacter junii, isolated in the same hospital, carrying the carbapenemase OXA-24/40 and displaying high resistance rates to carbapenems. By means of bioinformatics analysis we have also been able to characterize the mechanism by which this carbapenemase is horizontally transferred interspecies of Acinetobacter spp. The dissemination of carbapenemase OXA-24/40 between non-*baumannii* Acinetobacter species is concerning since it prevents the use of most β-lactam antibiotics in the fight against these resistant isolates.

## OBSERVATION

Species of the genus Acinetobacter can readily adapt to hospital environments and develop resistance to antibiotics. In this regard, the Acinetobacter calcoaceticus-Acinetobacter baumannii complex is of utmost importance as it is responsible for most human infections and hospital outbreaks caused by Acinetobacter spp (1). Genomic species such as Acinetobacter junii and Acinetobacter haemolyticus can also cause infection.

Carbapenems are β-lactam antibiotics considered “last-line agents” for the treatment of severe infections caused by Acinetobacter spp. Production of carbapenem-hydrolyzing class D ß-lactamases (CHDLs), such as OXA-24/40, hampers the use of these antibiotics to tackle not only multidrug-resistant A. baumannii infections (2), but also those caused by other usually susceptible Acinetobacter genomic species (3–5). In the Iberian Peninsula, distribution of *bla*_OXA-24/40_ has been endemic and associated with the clonal expansion of A. baumannii ST98 (6), unrelated to other clinically relevant Acinetobacter species such as A. junii and Acinetobacter nosocomialis. Hence, we aim here to describe for the first time the development of carbapenem-resistance in A. nosocomialis and A. junii isolates via acquisition of the *bla*_OXA-24/40_ gene and to characterize its genetic platform of horizontal gene transfer between different species of Acinetobacter.

Thus, during a Spanish multicenter study of Acinetobacter spp. in 2020, two strains of non-*baumannii* Acinetobacter genomic species, A. nosocomialis (HUAV-AN66) and A. junii (HUAV-AJ77), were isolated in the Microbiology Department at the Arnau de Vilanova University Hospital (Lleida). Ampicillin, piperacillin, ceftazidime, cefepime, imipenem, meropenem, ertapenem, tobramycin, gentamicin, ciprofloxacin, colistin, and tigecycline (Sigma) MICs were determined by reference broth microdilution and Etest (bioMérieux). EUCAST v11.0 clinical breakpoints and guidelines were used for interpretation.

To evaluate the antimicrobial resistance mechanisms as well as their genetic environment, purified genomic DNA from clinical isolates was sequenced in parallel using both short- (Illumina MiSeq platform, Illumina) and long-read (MinION, Oxford Nanopore Technologies) approaches, while A. baumannii transconjugants were sequenced only with long-read technologies, and then mapped against the reference isolates and visualized using UGENE (7). Low quality short reads were removed with Trimmomatic v.0.36, and low quality long reads with Porechop v0.2.4 (8). Likewise, *de novo* reads from isolates were assembled using the Unicycler v0.4.8 hybrid assembler. Plasmid sequences were annotated with Prokka v1.14.5 and antimicrobial resistance gene content was analyzed using ResFinder v4.1 (9).

Initial identification of A. nosocomialis HUAV-AN66 and A. junii HUAV-AJ77 clinical isolates by MALDI-TOF was later confirmed by Ribosomal Multilocus Typing (rMLST) prediction (10) from assembled genomes. Both isolates were confirmed as carbapenem-resistant, displaying imipenem, meropenem and ertapenem MICs ≥64 mg/L. They also showed co-resistance to penicillins and gentamicin, but exhibited susceptibility or borderline MICs to ceftazidime, tobramycin, ciprofloxacin, colistin and tigecycline (Table 1). The *bla*_OXA-24/40_ gene was the only gene associated with carbapenem resistance in both isolates, initially identified by automatized PCR and later confirmed by whole-genome sequencing (WGS).

**TABLE 1:** Bacterial strains used in the study and antibiotic susceptibility profiles

Strains	Description	MICs (mg/L)^*a*^											
		AMP	PIP	CAZ	FEP	IMI	MEP	ERT	TOB	GEN	CIP	COL	TGC
A. nosocomialis HUAV-AN66	Clinical strain. Donor of plasmids pHUAV/OXA-24/40 and pHUAV/AMG-R	≥2048	≥512	4	8	64	128	≥64	4	32	≤0.12	2	0.5
A. junii HUAV-AJ77	Clinical strain. Donor of plasmids pHUAV/OXA-24/40 and pHUAV/AMG-R	256	128	2	2	64	64	≥64	2	32	0.5	2	0.5
A. baumannii ATCC 19606 *pmrB* ^*A*^^227V^	Recipient plasmids pHUAV/OXA-24/40 and pHUAV/AMG-R. Colistin-resistant.	256	32	4	2	0.25	0.5	0.5	0.25	≤0.12	≤0.12	64	0.5
A. baumannii ATCC 19606 *pmrB*^*A*^^227V^/pHUAV/OXA-24/40 + pHUAV/AMG-R (HUAV-AN66)	Receptor of pHUAV/OXA-24/40 and pHUAV/AMG-R from A. nosocomialis	1024	256	4	2	64	64	≥64	2	16	≤0.12	64	0.5
A. baumannii ATCC 19606 *pmrB*^*A*^^227V/^pHUAV/OXA-24/40+ pHUAV/AMG-R (HUAV-AJ77)	Receptor of pHUAV/OXA-24/40 and pHUAV/AMG-R from A. junii	1024	256	4	4	64	64	≥64	2	16	≤0.12	128	0.25
A. baumannii ATCC 19606 *pmrB*^*A*^^227V^/pHUAV/AMG-R (HUAV-AN66)	Receptor of pHUAV/AMG-R from A. nosocomialis	256	32	4	2	0.25	0.25	1	2	16	≤0.12	64	0.5
A. baumannii ATCC 19606 *pmrB*^*A*^^227V^/pHUAV/AMG-R (HUAV-AJ77)	Receptor of pHUAV/AMG-R from A. junii	256	32	4	2	0.25	0.25	1	2	16	≤0.12	64	0.5
A. baumannii AbH12O-CU2	Clinical strain. Carrier of plasmid pMMCU2 (harbouring OXA-24/40)	1024	128	4	4	32	128	64	1	0.25	≤0.12	1	1

a AMP, ampicillin; PIP, piperacillin; CAZ, ceftazidime; FEP, cefepime; IMI, imipenem; MEP, meropenem; ERT, ertapenem; TOB, tobramycin; GEN, gentamicin; CIP, ciprofloxacin; COL, colistin and TGC, tigecycline.

**FIG 1:**
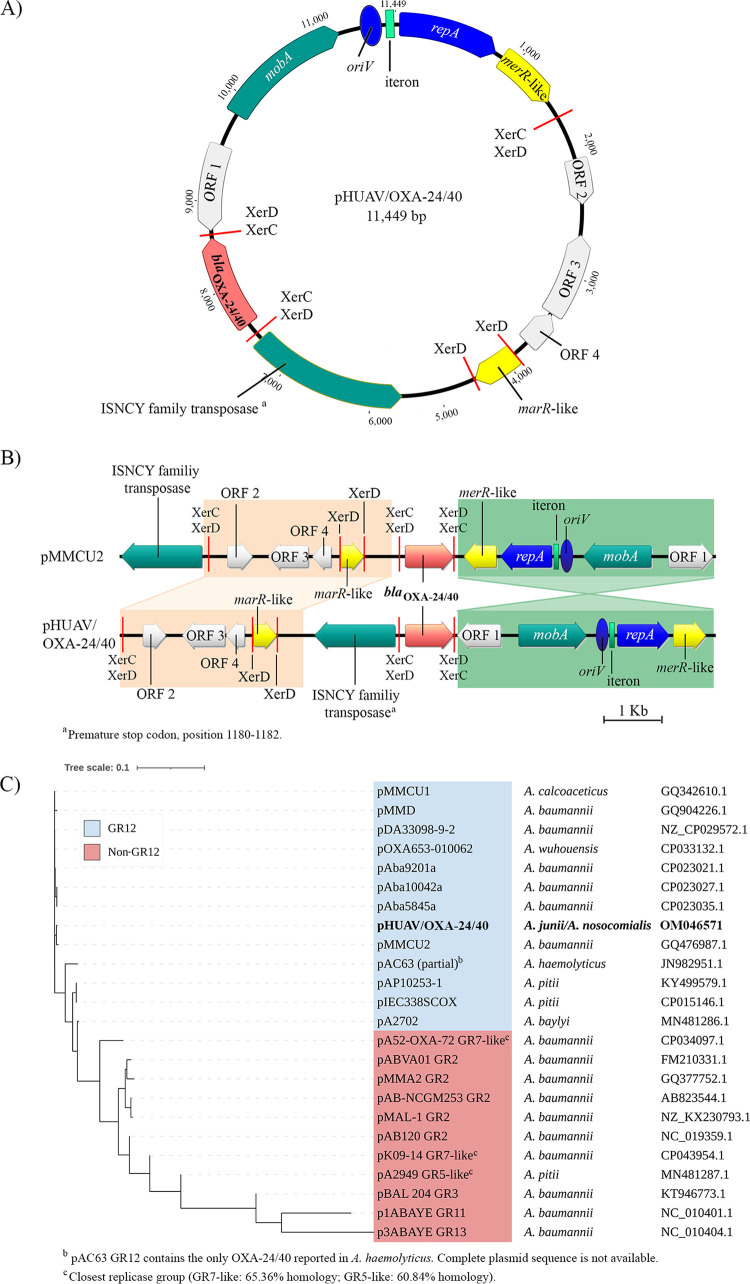
(A) Structure of the 11,449 pb GR12 pHUAV/OXA-24/40 plasmid obtained from the clinical strains A. nosocomialis HUAV-AN66 and A. junii HUAV-AJ77. All arrows represent length and transcription of the genes. *bla*_OXA-24/40_ gene is highlighted in red. Blue represents DNA replication; green, DNA transmission; yellow, antibiotic/metal resistance and gray, hypothetical CDS. (B) Linear comparison of plasmids pHUAV/OXA-24/40 and pMMCU2, harboring *bla*_OXA24/40_. XerC/XerD recombination sites are indicated. (C) Dendrogram of the sequenced plasmids harboring the *bla*_OXA-24/40_, constructed using the Mash distance method. The plasmids sharing the same replicase gene (*repA_AB*), and which hence are part of the same GR12, are highlighted in blue; plasmids belonging to other plasmid groups are highlighted in red. Plasmid names and accession numbers for the corresponding Acinetobacter species are shown on the right.

Characterization of *bla*_OXA-24/40_ genetic environment in both A. nosocomialis and A. junii isolates revealed a small plasmid of 11,449 bp, with 10 open reading frames (Fig. 1A and Table S1) and designated pHUAV/OXA-24/40. A second plasmid of 300,837 bp carrying the aminoglycoside resistance genes *aac(3′)-IIa*, *aph(6)-Id*, and *aph(3′')-Ib*, encoding aminoglycoside (mainly gentamicin, tobramycin and streptomycin) modifying enzymes (11), was also identified in both clinical isolates and designated pHUAV/AMG-R (Fig. S1 and Table S2).

*In silico* analysis showed that pHUAV/OXA-24/40 possessed a very similar environment to that of the previously identified pMMCU2 (GQ476987, identity >95%), displaying highly conserved genetic structures harboring the *bla*_OXA-24/40_ (Fig. 1B and Table 1). pMMCU2 was first detected by our group in one of the largest A. baumannii outbreaks so far, which occurred in 2008 in the 12 October Hospital, (Madrid, Spain) (9). During its course, A. baumannii AbH12O-A2 (main epidemic clone), A. baumannii AbH12O-CU2, and A. calcoaceticus AbH12O-CU1 were found to carry plasmids pMMA2(GQ377752.1), pMMCU2, and pMMCU1 (GQ342610) (5, 12), respectively. All of them encoded the *bla*_OXA-24/40_ gene flanked by XerC/XerD recombination sites (Fig. 1C). These inverted repeat sequences (XerC-6pb-XerD) are targeted by the chromosomal recombinases XerC and XerD and have been suggested to act by mobilizing the *bla*_OXA-24/40_ gene, thus facilitating its worldwide distribution (3, 13).

Horizontal transfer assays were then performed to assess the bacterial transmission of this resistance determinant. Briefly, the previously described colistin-resistant A. baumannii ATCC 19606 *pmrB*^A227V^ (recipient) (14) and HUAV-AN66/HUAV-AJ77 (donors) isolates were grown overnight in Luria Bertani (LB) broth and then mixed (ratio 2:1 and 1:10 for HUAV-AN66 and HUAV-AJ77, respectively) and cultured for 20h on 10 ml of LB and brought to a final volume of 3 ml. Serial dilutions were spread on agar plates containing 15 mg/L colistin and, as appropriate 10 mg/L meropenem and/or 10 mg/L gentamicin. Conjugation frequencies were estimated by counts on the growth plates. Transfer of *bla*_OXA-24/40_ was confirmed by PCR amplification of *bla*_OXA-24/40_-like (primers OXA24-Fw: GGTTAGTTGGCCCCCTTAAA/OXA24-Rv: AGTTGAGCGAAAAGGGGATT). A. baumannii receptor cells were confirmed by *bla*_OXA-51_-like PCR amplification (primers OXA51-Fw: TAATGCTTTGATCGGCCTTG/OXA51-Rv: TGGATTGCACTTCATCTTGG). Selected transconjugants and plasmids were also confirmed by further WGS, as previously commented.

In conjugation assays, plasmids pHUAV/OXA-24/40 and pHUAV/AMG-R were successfully transferred together from both A. nosocomialis HUAV-AN66 and A. junii HUAV-AJ77 to the receptor A. baumannii ATCC 19606 *pmrB*^A227V^, which displayed high carbapenem resistance (MIC ≥64 mg/L) and increased the MICs to gentamicin and tobramycin. In addition, transconjugants of A. baumannii ATCC 19606 *pmrB*^A227V^ carrying only the pHUAV/AMG-R plasmid were also obtained in conjugation assays with both clinical strains, increasing the MIC to gentamicin and tobramycin 128 and 8-fold, respectively (Table 1).

Self-transmissible plasmids by conjugation must encode a complete set of components required for transfer such as a relaxase (Mob protein), and the type IV secretion system (T4SS) (15–17). Here, we show that pHUAV/OXA-24/40 encodes the relaxase protein MobA, but no other conjugation-related genes, and is thus a mobilizable but not self-transmissible plasmid (Fig. 1A and Suplementary Table 1). These results are concordant with analysis performed by Salgado-Camargo et al., who found that most of A. baumannii plasmid lineages do not have the large sets of conjugation genes, such as T4SS (>10 genes) (13). Small plasmids that code for a relaxase rarely code for a type IV coupling protein (T4CP) (6%), whereas larger plasmids generally do (86%). Thus, mobilizable plasmids containing just a Mob protein need a coresident self-conjugative plasmid to be transmissible by conjugation from donor to recipient (18). The pHUAV/AMG-R plasmid, present in both clinical isolates, encodes a whole conjugative system: a replicase, a relaxase, a T4CP, and a T4SS (Dot/Icm conjugation system). This Dot/Icm conjugation machinery, previously found in Legionella pneumophila, is related to the Tra/Trb genes required for conjugation of self-transmissible IncI plasmids (19, 20) (Fig. S1 and Table S2).

Because pHUAV/AMG-R was shown to carry genes encoding plasmid conjugation functions, assays were then performed in order to test horizontal transfer efficiency of both plasmids. The results showed that pHUAV/AMG-R could be directly conjugated from A. nosocomialis to A. baumannii, with an efficiency of 1.10 × 10^−6^, while both plasmids simultaneously were conjugated with an efficiency of 8.24 × 10^−8^, respectively. Likewise, pHUAV/AMG-R or both plasmids were also conjugated from A. junii to A. baumannii with an efficiency of 4.06 × 10^−8^ and 6.01 × 10^−9,^ respectively. The pHUAV/OXA-24/40 plasmid was also able to mobilize alone to the recipient A. baumannii strain, although at lower rates than the both plasmids together: 2.70 × 10^−9^ from A. nosocomialis and 1.21 × 10^−9^ from A. junii, thus displaying a very low frequency of horizontal plasmid transfer.

The epidemiology of plasmid pHUAV/OXA-24/40 and context of *bla*_OXA-24/40_ were then assessed. The pHUAV/OXA-24/40 plasmid, along with all of the plasmids identified in AbH12O-CU1, AbH12O-CU2 and AbH12O-D A. baumannii isolates from the Madrid outbreak (12), belong to group 12 (GR12), according to the classification scheme proposed by Bertini et al. (PCR-based on replicon typing, AB-PBRT method) (21), and show identical *rep* gene sequences (*repA_AB*). The spread of OXA-24/40 in the Iberian Peninsula has mainly been linked to the success of the A. baumannii ST98 clone; however, circulation of common *bla*_OXA-24/40_-carrying plasmids has probably also played an important role in the dissemination of this carbapenemase (3). Analysis of the distribution of these plasmids in GenBank databases showed that 10-kb GR12 plasmids harboring *bla*_OXA-24/40_, displaying high identity with pHUAV/OXA-24/40 (>98%), besides to being localized mostly in A. baumannii, are the main responsible for transmission of this carbapenemase gene between different Acinetobacter species, including (in addition to A. junii and A. nosocomialis described here) *A. haemolyticus*, A. calcoaceticus, Acinetobacter pittii, Acinetobacter baylyi, and Acinetobacter *wuhouensis* (Fig. 1C). Thus, 10-kb GR12 plasmids seem to be the most important drivers of transmission of carbapenem resistance via acquisition of OXA-24/40 among isolates of non-*baumannii* Acinetobacter species.

Last, phylogenetic analysis was performed using plasmid sequences harboring *bla*_OXA-24/40_-like genes (extracted from GenBank); p1ABAYE and p2ABAYE plasmids, which did not harbor these genes, were used as an outgroup. Phylogenetic distance was calculated using the alignment-free MASH method, based on k-mers, allowing complete genome analysis. Finally, bootstrapping (1000 re-samples) was used yield robust confidence levels (22).

The phylogenetic analysis showed that plasmids closely related to pMMCU2 (phylogenetic distance <0.018) -all belonging to the GR12 and carrying *bla*_OXA-24/40_-like genes (Fig. 1C and Fig. S2)- have been identified in Portugal, Brazil, China, and Mexico. This indicates that in addition to serving as platform for transmission between Acinetobacter species, 10-kb GR12 plasmids also play a role in the international spread of the carbapenemase OXA24/40.

In conclusion, the carbapenemase OXA24/40 was identified, for the first time, in A. nosocomialis and A. junii and conferred high resistance to carbapenems. In both isolates, OXA-24/40 was located in the pHUAV/OXA-24/40 plasmid and linked to plasmid pMMCU2, previously isolated from A. baumannii. GR12 plasmids harboring *bla*_OXA-24/40_ represent a risk for the expansion of this carbapenem-resistance mechanism among non-*baumannii* Acinetobacter species.

### Nucleotide accession number.

The complete sequence of plasmids pHUAV/OXA-24/40 and pHUAV/AMG-R have been deposited in GenBank database under accession number OM046571 and OM046572, respectively. The raw reads of both complete genomes can be found at PRJNA731124.
