# Supplementary material for: Carbapenem Resistance in Acinetobacter nosocomialis and Acinetobacter junii Conferred by Acquisition of blaOXA-24/40 and Genetic Characterization of the Transmission Mechanism between Acinetobacter Genomic Species
Source: Microbiol Spectr. 2022 Feb 9;10(1):e02734-21. doi: 10.1128/spectrum.02734-21 (PMC8826734; doi:10.1128/spectrum.02734-21)

Supplementary material

Title: Carbapenem resistance in *Acinetobacter nosocomialis* and *Acinetobacter junii* conferred by acquisition of *bla*$_{OXA-24/40}$ and genetic characterization of the transmission mechanism between *Acinetobacter* genomic species.

Authors: Cristina Lasarte-Monterrubio, Paula Guijarro-Sánchez, Alba Bellés, Juan Carlos Vázquez-Ucha, Jorge Arca-Suárez, Carlos Fernandez-Lozano, German Bou and Alejandro Beceiro on behalf of the Spanish National Study *Acinetobacter* spp. 2020 Group.

Table S1. Genetic content of plasmid pHUAV/OXA-24/40.

Nucleotide position	Strand	Gene/Feature	Properties and/or putative function
1-888	+	*repA*	Replicase, replication initiator protein
929-1522	+	*merR*-like	Mediates the induction of mercury resistance operon
1674-1700	N.A.	XerC/XerD	Recombination sites of XerC and XerD recombinases
2086-2484	+	ORF2	Hypothetical protein
2760-3494	-	ORF3	Hypothetical protein
3513-3833	-	ORF4	Hypothetical protein
3982-3992	N.A.	XerD	Recombination site of XerD recombinase
3981-4394	+	*marR*-like	Transcription factor, implicated in antibiotic resistance
4395-4405	N.A.	XerD	Recombination site of XerD recombinase
6038-7411	-	ISNCY family transposase	Inactivated transposase with Stop codon in position 6430
6495-6522	N.A.	XerC/XerD	Recombination sites of XerC and XerD recombinases
7785-8612	+	*bla*$_{OXA-24/40}$	Carbapenemase, carbapenem hydrolysis
8681-9439	-	ORF1	Hypothetical protein
9743-10912	+	*mobA*	Relaxase
11093-11293	-	*oriV*	Origin of vegetative replication
11310-11405	-	Iteron	Repeated DNA sequence, regulates the synthesis of the RepA protein

N.A.: Not applicable

Table S2. Relevant genetic content of plasmid pHUAV/AMG-R.

Nucleotide position	Strand	Gene/Feature	Properties and/or putative function
47867-50872	+	*dotA*	Type IV secretion system protein DotA, critical for efficient efector delivery
59661-61190	-	*dotM*	Type IV secretion system protein IcmP/DotM
66507-67802	-	*dotB*	Type IV secretion system protein DotB
67830-68639	-	*dotC*	Type IV secretion system protein DotC
68680-69123	-	*dotD*	Type IV secretion system protein DotD/TraH
71730-72413	+	*dotI*	Putative type IV secretion protein IcmL/DotI
72427-73374	+	*dotH*	Putative type IV secretion protein IcmK/DotH
73396-74907	+	*dotG*	Type IV secretion system protein IcmE/DotG
75805-76380	+	putative type IV secretion protein	Putative relaxase
79230-82412	+	*dotO*	Type IV secretion system protein IcmB/DotO
82535-85327	+	*dotL*	Type 4 coupling protein (T4CP) IcmO/DotL
100061-100921	-	*aac(3)-IIa*	Aminoglycoside N-acetyltransferase AAC(3)-IId
103930-104733	+	*aph(3'')-Ib*	StrA, aminoglycoside phosphotransferase
104733-105569	+	*aph(6)-I*	StrB, APH(6)-Id, streptomycin(6) phosphotransferase
108829-109131	+	*relE/parE*	Type II toxin-antitoxin system RelE/ParE family toxin
111134-112609	+	*msr(E)*	ABC-F type ribosomal protection protein Msr(E), macrolide resistance
112665-113549	+	*mph(E)*	Mph(E) family macrolide 2'-phosphotransferase
282904-284694	-	*rep*-like	Putative replicase derived from pALWED1.1 megaplasmid

Figure S1. Structure of the 300,837 pb pHUAV/AMG-R plasmid obtained from the clinical strains *A. nosocomialis* HUAV-AN66 and *A. junii* HUAV-AJ77. All arrows represent length and transcription of the genes. Resistance genes (aminoglycoside and macrolides) are highlighted in red, green represents type II toxin-antitoxin and blue represents DNA replication and conjugation.

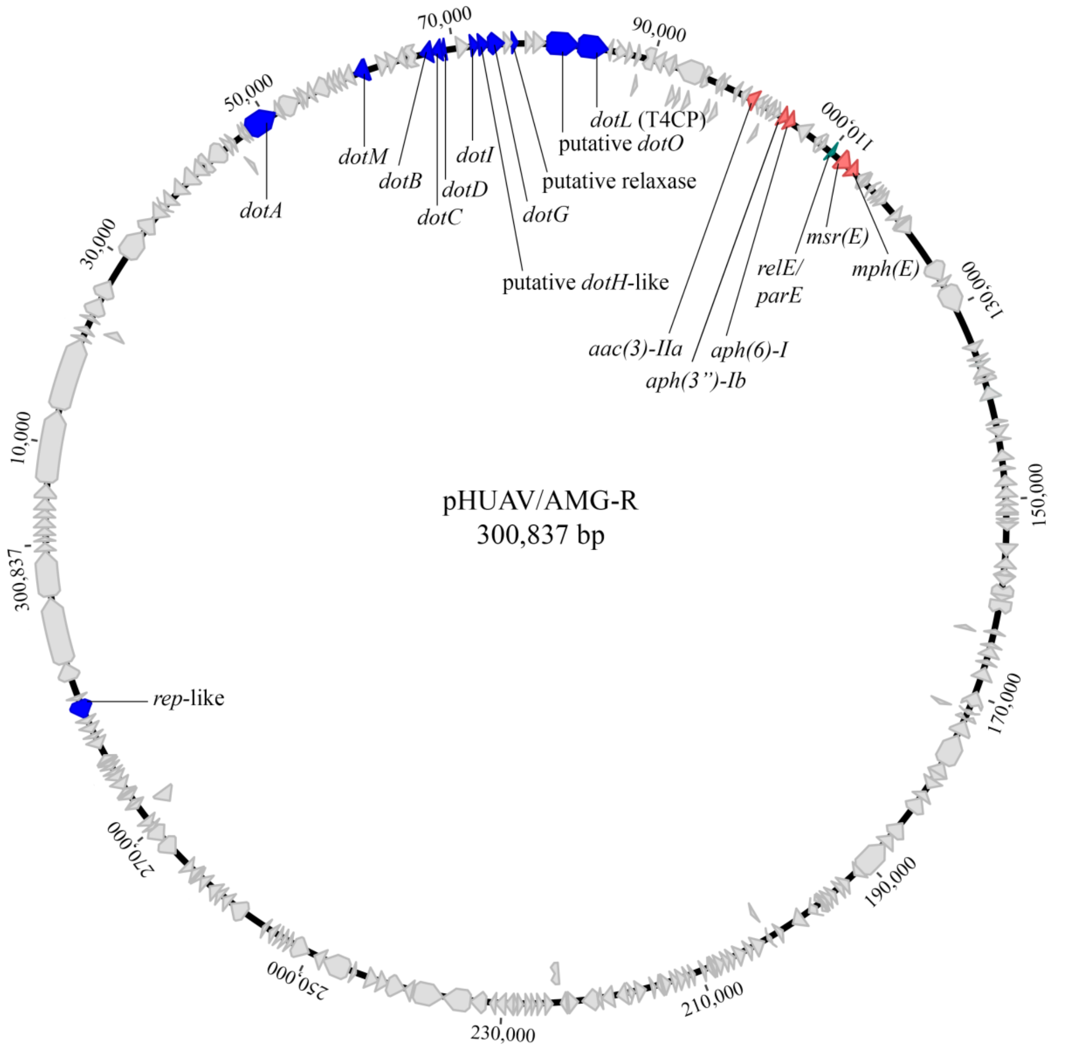

Figure S2. International distribution of plasmids 10-kb GR-12 (replicase *repA_AB*, GenBank: ACT83384.1) carrying $bla_{OXA-24/40}$ identified in *Acinetobacter* spp.

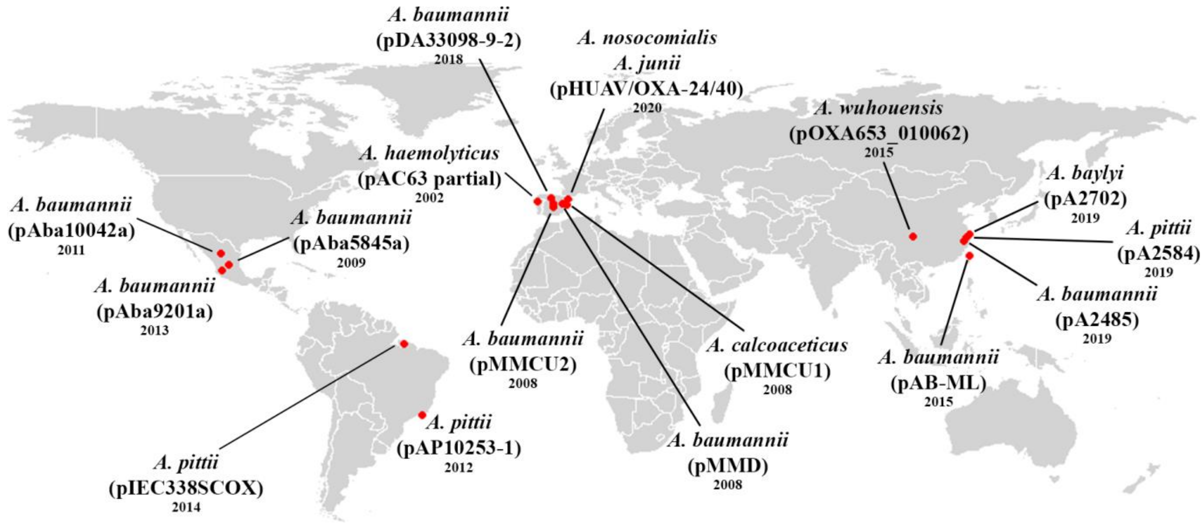

Supplement: SUPPLEMENTAL FILE 1 — Supplemental material. Download SPECTRUM02734-21_Supp_1_seq1.pdf, PDF file, 0.5 MB [file spectrum02734-21_supp_1_seq1.pdf]
